# Factors affecting serum albumin in the perioperative period of colorectal surgery: a retrospective study

**DOI:** 10.1186/s13104-015-1632-8

**Published:** 2015-11-03

**Authors:** Akihiro Sonoda, Shun Ohnishi, Shoji Nakao, Yoshitaka Iwashita, Naomi Hashimoto, Kazuhisa Ishida, Yuki Kondo, Yoichi Ishitsuka, Tetsumi Irie

**Affiliations:** Department of Pharmacy, Izumi Regional Medical Center, 4513 Akasegawa, Akune, Kagoshima 899-1611 Japan; Department of Gastroenterology, Izumi Regional Medical Center, 4513 Akasegawa, Akune, Kagoshima 899-1611 Japan; Department of Clinical Chemistry and Informatics, Graduate School of Pharmaceutical Sciences, Kumamoto University, 5-1 Oe-honmachi, Chuo-ku, Kumamoto, 862-0973 Japan; Center for Clinical Pharmaceutical Sciences, Faculty of Pharmaceutical Sciences, Kumamoto University, 5-1 Oe-honmachi, Chuo-ku, Kumamoto, 862-0973 Japan

**Keywords:** C-reactive protein, Perioperative period, Serum albumin

## Abstract

**Background:**

Albumin is considered a negative acute-phase protein because its concentration decreases during injury and sepsis. Hypoalbuminemia is a risk factor for mortality, postoperative complications, and prolonged hospital stay. The magnitude of the systemic inflammatory response during the perioperative period, as indicated by the acute-phase proteins—C-reactive protein (CRP) in particular—, may help identify the risk of postoperative infectious complication. The correlation between serum albumin and CRP with gastrointestinal cancer has been reported. However, it is unclear whether antecedent CRP could be utilized to predict future hypoalbuminemia in the perioperative period in colorectal surgery. The primary endpoint of this study was to reveal that antecedent CRP could be utilized to predict future hypoalbuminemia in the perioperative period of colorectal surgery.

**Methods:**

Thirty-seven patients who underwent elective open colorectal surgery were included in this study. Correlations between preoperative CRP and serum albumin on postoperative day (POD) 3, between preoperative CRP and serum albumin on POD 7 and between CRP on POD 3 and serum albumin on POD 7 were examined. Relationships between preoperative CRP and hypoalbuminemia on POD 3, between preoperative CRP and hypoalbuminemia on POD 7 and between CRP on POD 3 and hypoalbuminemia on POD 7 were examined by receiver operating characteristic analysis.

**Results:**

Three-quarters of patients were older than 65 years of age. Significant correlations were observed between preoperative CRP and serum albumin on POD 3 (*p* = 0.023), between preoperative CRP and serum albumin on POD 7 (*p* = 0.023) and between CRP on POD 3 and serum albumin on POD 7 (*p* < 0.001). The area under the receiver operating characteristic curve of CRP on POD 3 to development of hypoalbuminemia on POD 7 was 0.833 (95 % CI 0.679–0.987) with an optimal threshold of 12.43 mg/dL, sensitivity 75 % and specificity 80 %.

**Conclusions:**

The present study revealed that antecedent CRP was associated with future serum albumin. Additionally, CRP on POD 3 could be useful in predicting the development of hypoalbuminemia on POD 7. This result suggests that CRP on POD 3 may be a valuable indicator for early nutritional intervention.

## Background

Colorectal surgery has traditionally been associated with significant morbidity and prolonged hospital stay [1–4]. Overall complication rates have been reported to be 26–35 % [1, 3, 4]. Infectious complications, in particular, represent a major cause of morbidity and mortality after colorectal surgery [4, 5].

Albumin is considered a negative acute-phase protein because its concentration decreases during injury and sepsis. The rate of loss of albumin to the tissue spaces (measured as transcapillary escape rate) rises by more than 300 % in patients with septic shock [6, 7]. Hypoalbuminemia is a risk factor for mortality and postoperative complications [8–13]. Therefore, nutritional control has been an important focus of perioperative management [14].

The magnitude of the systemic inflammatory response during the perioperative period, as indicated by the acute-phase proteins—C-reactive protein (CRP) in particular—may help to identify the risk of a postoperative infectious complication [4, 15–21].

The correlation between serum albumin and CRP with gastrointestinal cancer has been reported [22, 23]. However, it is unclear whether antecedent CRP could be used to predict future hypoalbuminemia in the perioperative period of colorectal surgery.

The primary endpoint of this study was to reveal whether antecedent CRP could be used to predict future hypoalbuminemia in the perioperative period of colorectal surgery. The secondary endpoint was to clarify the relationship between CRP on postoperative day (POD) 3 and postoperative infectious complications.

## Methods

### Study design

This retrospective study included patients who had been admitted for elective open colorectal surgery from July 2011 to March 2013 at the Izumi Regional Medical Center. The following patient data were collected from medical charts: sex, age, albumin administration in the postoperative period, body mass index (BMI), type of surgery, tumor site, American Joint Committee on Cancer (AJCC) tumor-node-metastasis (TNM) staging, depth of tumor invasion, lymph node involvement, and postoperative oral intake. The tumors were staged according to the TNM criteria [24].

The following laboratory data were determined preoperatively and on PODs 3 and 7: serum albumin, CRP, aspartate aminotransferase (AST), alanine aminotransferase (ALT), gamma-glutamyl transpeptidase (γ-GTP), lactate dehydrogenase (LDH), alkaline phosphatase (ALP), serum creatinine (Scr), blood urea nitrogen (BUN), hemoglobin (Hb), and white blood cell (WBC) count. Serum levels of albumin (normal range 4.0–5.0 g/dL) and CRP (normal range 0–0.3 mg/dL) were measured using the bromocresol green dye-binding method and turbidimetric assay with an autoanalyzer (Hitachi 7180; Hitachi, Tokyo, Japan).

Patients underwent mechanical bowel preparation with 2 L of polyethylene glycol electrolyte solution (Niflec; Ajinomoto Pharma, Tokyo, Japan). Prophylactic cefmetazole was administered from the day of the surgery (3 g/day) to POD 2 (2 g/day on POD 1 and POD 2). The study protocol was approved by the ethics committee of Izumi Regional Medical Center (approval number 20130812-1).

### Examination of factors affecting perioperative serum albumin with colorectal surgery

Preoperative hypoalbuminemia is a risk factor for postoperative complications [8, 11, 12]. Platt et al. provided data WBC, CRP, and albumin concentrations on preoperative and PODs 1–7 in 454 patients undergoing surgery for colorectal cancer, of whom 104 developed infectious complications. Results demonstrated that CRP measurements on POD 3 could accurately predict infectious complications, including anastomotic leak, after resection for colorectal cancer [4]. The average time to development of an infectious complication, including an anastomotic leak, was between 6 and 8 days postoperatively [4]. These results demonstrated the utility of factors affecting serum albumin on the preoperative day and PODs 3 and 7.

Moyes et al. reported that preoperative elevated modified Glasgow Prognostic Score predicts postoperative infectious complications in patients undergoing potentially curative resection for colorectal cancer [25]. Therefore, the independent variables with a possible effect on serum albumin were chosen by referring to this report [25]. The dependent variable was serum albumin and the independent variables were CRP, sex (male, 1; female, 0), age, albumin administration on the postoperative day (yes, 1; no, 0), tumor site (rectum, 1; colon, 0), AJCC TNM cancer stage (I, 1; II, 2; III, 3; IV, 4), depth of tumor invasion (T1, 1; T2, 2; T3, 3; T4, 4), lymph node involvement (N0, 0; N1, 1; N2, 2), BMI, postoperative oral intake (bad, 1; good, 0), AST, ALT, γ-GTP, LDH, ALP, Scr, BUN, Hb, and WBC count. Postoperative oral intake was used as an independent variable only on POD 7. Postoperative albumin administration was used as an independent variable on PODs 3 and 7.

### Correlations between antecedent CRP and future serum albumin

We examined correlations between preoperative CRP and serum albumin on POD 3, between preoperative CRP and serum albumin on POD 7 and between CRP on POD 3 and serum albumin on POD 7.

### Relationships between antecedent CRP and future hypoalbuminemia

By receiver operating characteristic (ROC) analysis [4], we examined relationships between preoperative CRP and hypoalbuminemia on POD 3, between preoperative CRP and hypoalbuminemia on POD 7 and between CRP on POD 3 and hypoalbuminemia on POD 7. Hypoalbuminemia was defined as serum albumin ≤3.0 g/dL [14, 26].

### Relationship between CRP on POD 3 and postoperative infectious complications

Patients were assessed for the following infectious complications: wound infection, intra-abdominal abscess, anastomotic leak, pneumonia and septicemia [25]. The criteria used to define infectious complications were taken from the methods reported by Moyes et al. [25]: (1) wound infection was defined as the presence of pus, either discharged spontaneously or requiring drainage. Wound infection included a subgroup of patients who developed perineal infection after abdominoperineal resection of the rectum. (2) Intra-abdominal abscess was verified by either surgical drainage or by ultrasonographically guided aspiration of pus. (3) Anastomotic leakage was defined as radiologically verified fistula to bowel anastomosis or diagnosed by repeat laparotomy. (4) Pneumonia was defined as a positive chest radiograph and requirement for antibiotic treatment. (5) Septicemia was defined by clinical symptoms combined with a positive blood culture. To reveal the relationship between CRP on POD 3 and postoperative infectious complications, the diagnostic accuracy of CRP was assessed by ROC analysis [4].

### Statistical analysis

Multiple regression analysis with stepwise variable selection was used to examine the factors affecting preoperative day and PODs 3 and 7 serum albumin with significance level of entering a selection at *p* < 0.05 and of keeping a selection at *p* < 0.10 [27]. The significance level for keeping an independent variable in the final model was set at 0.01.

The relationships between antecedent CRP and future hypoalbuminemia were examined by ROC analysis [4]. The relationships between CRP on POD 3 and postoperative infectious complications were performed using ROC analysis [4]. The area under the ROC curve (AUC) results were considered excellent for AUC values between 0.9 and 1, good for AUC values between 0.8 and 0.9, fair for AUC values between 0.7 and 0.8, poor for AUC values between 0.6 and 0.7 and failed for AUC values between 0.5 and 0.6 [28]. Statistical analysis was performed using Excel 2010 (Microsoft Corp., Redmond, WA, USA) with the add-in software Ekuseru-Toukei 2012 (Social Survey Research Information Co., Ltd., Tokyo, Japan). Additionally, EZR (Saitama Medical Center, Jichi Medical University, Japan), which is a graphical user interface for R [29] (The R Foundation for Statistical Computing, Vienna, Austria) was used for ROC analysis only.

## Results

### Patient characteristics

Patient characteristics are presented in Table 1. Three-quarters of patients were older than 65 years of age. Laboratory values revealed no severe perioperative liver or kidney dysfunction.

**Table 1:** Characteristics of 37 patients who underwent colorectal surgery

Patients characteristics (n = 37)	Values
Sex, no (%)	
Male/female	19 (51):18 (49)
Age (years)	77 (38–86)
BMI (kg/m^2^)	22 (15.8–31)
Tumor site, no. (%)	
Colon/rectum	25 (68):12 (32)
Type of surgery, no. (%)	
Colectomy	25 (68)
Anterior resection	9 (24)
Abdominoperineal resection of rectum	2 (5)
Hartmann procedure	1 (3)
TNM staging, no. (%)	
Stage I	3 (8)
Stage II	14 (38)
Stage III	17 (46)
Stage IV	3 (8)
Laboratory values in preoperative period	
Serum albumin (g/dL)	4.1 (2.5–5.0)
CRP (mg/dL)	0.31 (0.03–16.67)
AST (IU/L)	21 (10–37)
ALT (IU/L)	17 (6–46)
γ-GTP (IU/L)	22 (8–123)
LDH (IU/L)	186 (124–500)
ALP (IU/L)	257 (122–679)
Serum creatinine (mg/dL)	0.64 (0.42–1.42)
BUN (mg/dL)	13.5 (5.8–40.8)
WBC (/μL)	6100 (2400–16500)
Hemoglobin (g/dL)	10.7 (5.4–16.6)

Quantitative variables are expressed as medians (minimum–maximum). Qualitative variables are expressed as absolute numbers (percentages)

*ALP* alkaline phosphatase, *ALT* alanine transaminase, *AST* aspartate transaminase, *BMI* body mass index, *BUN* blood urea nitrogen, *CRP* C-reactive protein, *γ-GTP* gamma glutamyl transpeptidase, *LDH* lactate dehydrogenase, *WBC* white blood cell

### Postoperative infectious complications

Postoperative complications are presented in Table 2. Twelve (32 %) patients experienced postoperative complications, and nine (24 %) experienced only infectious complications. The most serious infectious complication was anastomotic leak. The median time to development of an infectious complication was 5 postoperative days.

**Table 2:** Postoperative complications after colorectal surgery

Postoperative complications	Number	Percentage
Infectious complications	9	24
Anastomotic leak	1	3
Wound infection	2	5
Intra-abdominal abscess	2	5
Pneumonia	4^a^	10
Ileus	2	5
Cardiac complications	1	3
All complications	12	32
Mortality	0	0

^a^2 patients with ileus

### Factors affecting perioperative serum albumin with colorectal surgery

In the preoperative period, CRP and BUN were effective variables. CRP was significant (*p* < 0.01), and the partial correlation coefficient was −0.497 (Table 3).

**Table 3:** Variables identified as predicting serum albumin in the perioperative period of colorectal surgery

Point	Variable	Regression coefficient	Standard error	Standardized regression coefficient	Partial correlation coefficient	*P*
Preoperative period	Constant	4.676	0.254			<0.001
	CRP	−0.078	0.026	−0.468	−0.497	0.005
	BUN	−0.037	0.015	−0.393	−0.434	0.016
POD 3	Constant	2.379	0.376			<0.001
	Hb	0.119	0.035	0.617	0.532	0.002
	CRP	−0.022	0.008	−0.416	−0.439	0.012
	Albumin administration	0.305	0.133	0.374	0.387	0.029
	Lymph node involvement	0.182	0.085	0.311	0.366	0.040
	Scr	−0.529	0.279	−0.262	−0.327	0.068
	Tumor site	−0.264	0.141	−0.332	−0.324	0.070
POD 7	Constant	1.912	0.388			<0.001
	Hb	0.107	0.032	0.501	0.506	0.002
	CRP	−0.024	0.008	−0.429	−0.457	0.007
	γGTP	−0.006	0.002	−0.399	−0.424	0.012
	Depth of tumor invasion	0.162	0.085	0.268	0.319	0.066

*BUN* blood urea nitrogen, *CRP* C-reactive protein, *γ-GTP* gamma glutamyl transpeptidase, *Hb* hemoglobin, *POD* postoperative day

On POD 3, Hb, CRP, albumin administration on the postoperative day, lymph node involvement, SCr, and tumor site were effective variables. Hb was significant (*p* < 0.01), and the partial correlation coefficient was 0.532 (Table 3).

On POD 7, Hb, CRP, γ-GTP, and depth of tumor invasion were effective variables. Hb and CRP were significant (*p* < 0.01), and partial correlation coefficients were 0.506 and −0.457, respectively (Table 3).

### Correlations between antecedent CRP and future serum albumin

Significant correlations were observed between preoperative CRP and serum albumin on POD 3 (*p* = 0.023), between preoperative CRP and serum albumin on POD 7 (*p* = 0.023) and between CRP on POD 3 and serum albumin on POD 7 (*p* < 0.001) (Table 4).

**Table 4:** Correlations between antecedent CRP and future serum albumin in the perioperative period of colorectal surgery

Variable	Correlation coefficient	*P*
CRP in preoperative period and serum albumin on POD 3	−0.3742	0.0225
CRP in preoperative period and serum albumin on POD 7	−0.3723	0.0233
CRP on POD 3 and serum albumin on POD 7	−0.5447	0.0005

*CRP* C-reactive protein, *POD* postoperative day

### Relationship between antecedent CRP and future hypoalbuminemia

**Fig. 1:**
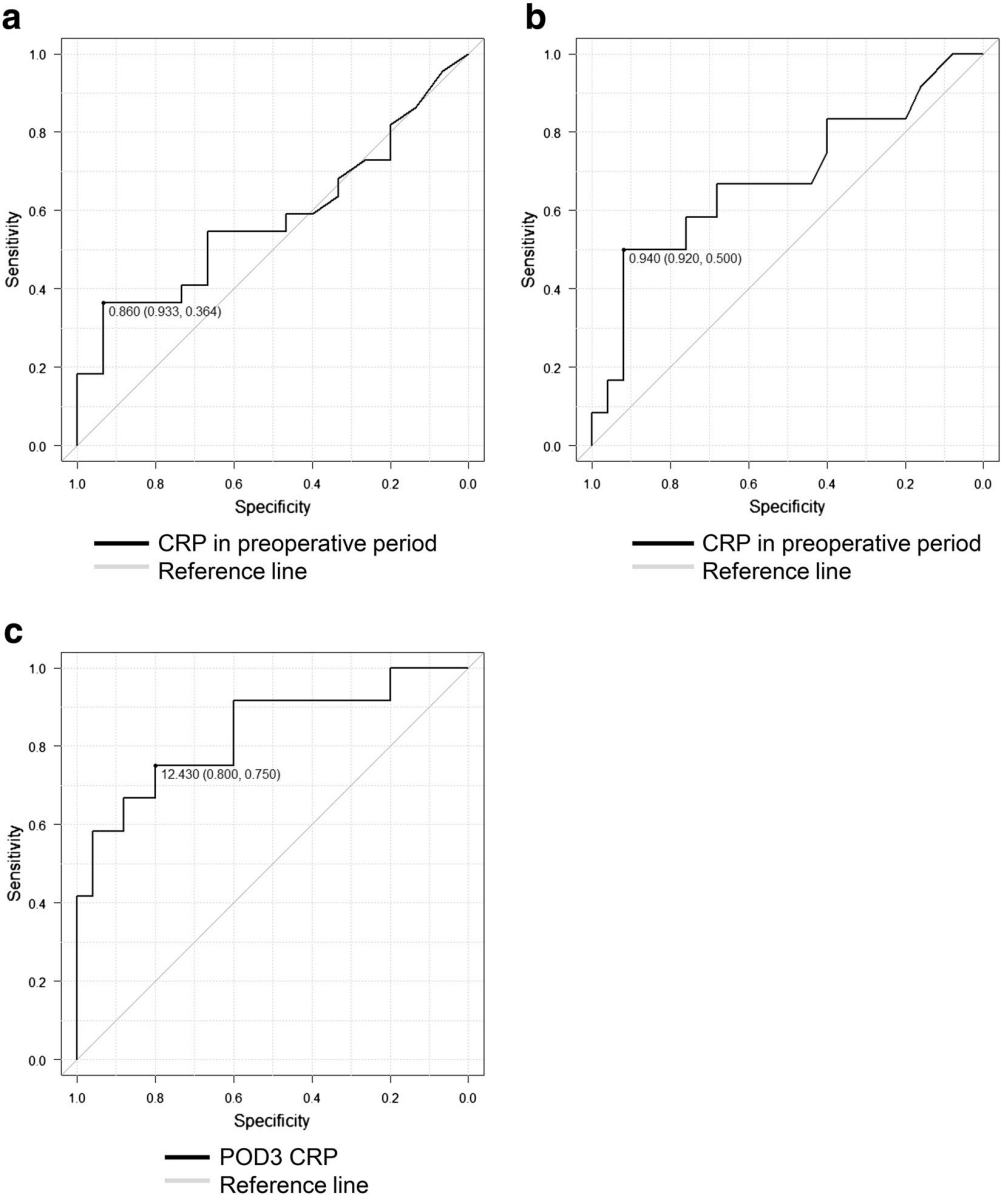
Diagnostic accuracy of antecedent CRP with regard to development of future hypoalbuminemia. **a** AUC of CRP in preoperative period to development of POD 3 hypoalbuminemia was 0.579 (95 % CI 0.392–0.766) with an optimal threshold of 0.86 mg/dL, sensitivity 36.4 % and specificity 93.3 %. **b** AUC of CRP in preoperative period to development of hypoalbuminemia on POD 7 was 0.683 (95 % CI 0.481–0.886) with an optimal threshold of 0.94 mg/dL, sensitivity 50 % and specificity 92 %. **c** AUC of CRP on POD 3 to development of hypoalbuminemia on POD 7 was 0.833 (95 % CI 0.679–0.987) with an optimal threshold of 12.43 mg/dL, sensitivity 75 % and specificity 80 %. *AUC* the area under the receiver operating characteristic curve, *CRP* C-reactive protein, *POD* postoperative day

The AUC of CRP in the preoperative period to the development of hypoalbuminemia on POD 3 was 0.579 (95 % CI 0.392–0.766) with an optimal threshold of 0.86 mg/dL, sensitivity of 36.4 % and specificity of 93.3 % (Fig. 1), and the diagnostic accuracy resulted as failed. The AUC of CRP in preoperative period to the development of hypoalbuminemia on POD 7 was 0.683 (95 % CI 0.481–0.886) with an optimal threshold of 0.94 mg/dL, sensitivity of 50 % and specificity of 92 % (Fig. 1) and the diagnostic accuracy was poor. The AUC of CRP on POD 3 to development of hypoalbuminemia on POD 7 was 0.833 (95 % CI 0.679–0.987) with an optimal threshold of 12.43 mg/dL, sensitivity of 75 % and specificity of 80 % (Fig. 1), and the diagnostic accuracy was good.

### Relationships between CRP on POD 3 and postoperative infectious complications

**Fig. 2:**
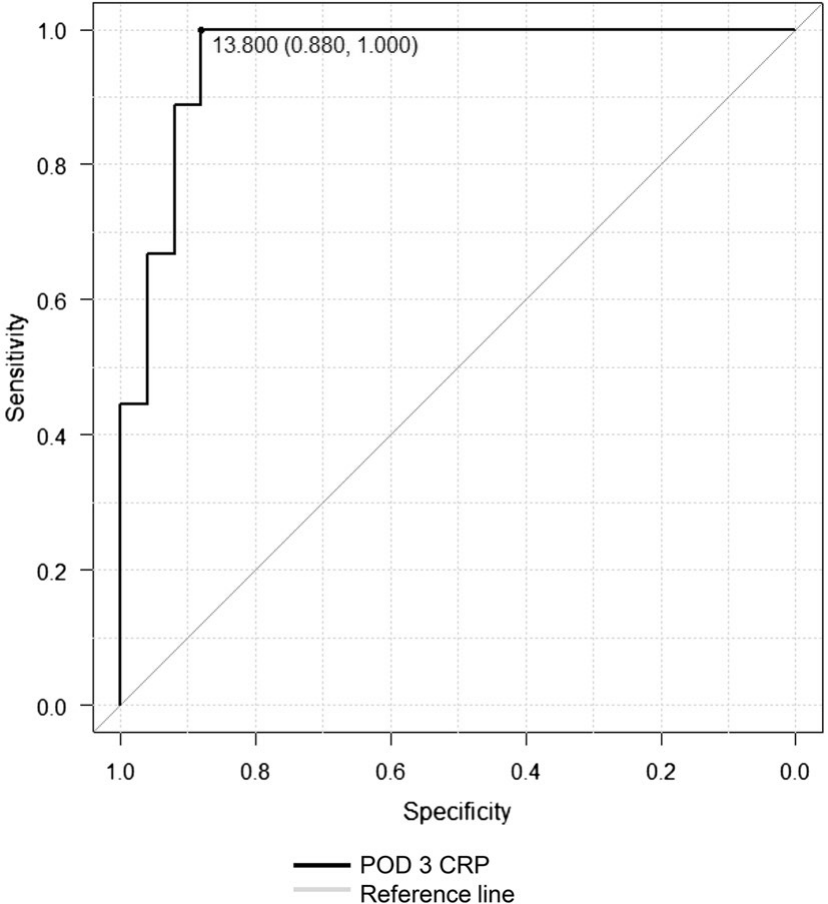
Diagnostic accuracy of CRP on POD 3 with regard to development of infective complications after colorectal surgery. AUC of CRP on POD 3 was 0.96 (95 % CI 0.902–1) with an optimal threshold of 13.8 mg/dL, sensitivity 100 % and specificity 88 %. *AUC* the area under the receiver operating characteristic curve, *CRP* C-reactive protein, *POD* postoperative day

The AUC of CRP on POD 3 was 0.96 (95 % CI 0.902–1) with an optimal threshold of 13.8 mg/dL, sensitivity of 100 % and specificity 88 % (Fig. 2), and the diagnostic accuracy was excellent.

## Discussion

In the present study, we examined whether antecedent CRP could be used to predict future hypoalbuminemia in the perioperative period of colorectal surgery. The main finding is that CRP on POD 3 may be of use in predicting the development of hypoalbuminemia on POD 7 (Fig. 1).

Three-quarters of patients were older than 65 years of age in the present study (Table 1). We searched for similar studies that evaluated infectious complications of colorectal surgery and found that 67 % of the patients in the study by Moyes et al. [25] were over 65 years of age, which is similar to the 67 % in Platt’s report [4], suggesting that the population in the present study is similar to the population in previous reports.

Twelve (32 %) patients experienced postoperative complications, and nine (24 %) experienced only infectious complications in the present study (Table 2). Overall complication rates have been reported to be 26–35 % in colorectal surgery [1, 3, 4]. Infectious complication rates have been reported to be 15–42 % in colorectal surgery [4, 25, 30, 31]. Therefore, the rates of all complications and infectious complications in the present study are similar to those in previous reports.

The correlation between serum albumin and CRP with gastrointestinal cancer has been reported previously [22, 23]. In present study, correlations were observed between serum albumin and CRP preoperatively (*p* < 0.01) and between serum albumin on POD 3 and CRP on POD 3 (*p* = 0.012) and between serum albumin on POD 7 and CRP on POD 7 (*p* < 0.01) (Table 3) in stepwise multiple regression analysis. These findings suggest that CRP has the greatest association with serum albumin, and concur with the results of other related reports.

Hypoalbuminemia is a risk factor for mortality and postoperative complications [8–13]. Therefore, the identification of a predictor of hypoalbuminemia may be clinically significant. In present study, significant correlations were observed between CRP in preoperative period and serum albumin on POD 3 (*p* = 0.023), between CRP in the preoperative period and serum albumin on POD 7 (*p* = 0.023) and between CRP on POD 3 and serum albumin on POD 7 (*p* < 0.001) (Table 4). Additionally, the AUC of CRP on POD 3 to the development of hypoalbuminemia on POD 7 was 0.833 (95 % CI 0.679–0.987) with an optimal threshold of 12.43 mg/dL, sensitivity 75 % and specificity 80 % (Fig. 1), suggesting that CRP on POD 3 could be useful in predicting the development of hypoalbuminemia on POD 7. Therefore, CRP on POD 3 may be valuable for the indicator of early nutritional intervention.

We consider that hypoalbuminemia resulted from increased CRP, which can be explained by the following: inflammatory cytokines decrease the synthesis of constitutive proteins, such as serum albumin, and increase its degradation [7]. They also promote capillary permeability and leakage of serum albumin into the extravascular space [7]. Because CRP is affected by increased interleukin-6 during acute inflammation, a decrease in serum albumin occurs with increased CRP [32].

The clinical utility of postoperative CRP has been reported [4, 33]. In particular, a large study (n = 454) by Platt et al. showed that CRP was a predictor of postoperative infectious complications after curative resection in patients with colorectal cancer and that postoperative measurement of CRP on POD 3 was clinically useful in predicting surgical site infectious complications, including anastomotic leak [4]. In that study, the AUC of CRP on POD 3 was 0.8 (*p* < 0.001) and the optimal cutoff value was 17 mg/dL, and the AUC of serum albumin on POD 3 was 0.68 (*p* < 0.001) and the optimal cutoff value was 2.5 g/dL. The diagnostic accuracy for postoperative infectious complications of CRP on POD 3 was better than that of serum albumin on POD 3 [4]. In the present study, the AUC of CRP on POD 3 with regard to development of infective complications after colorectal surgery was 0.96 (95 % CI 0.902–1) with an optimal threshold of 13.8 mg/dL, sensitivity 100 % and specificity 88 % (Fig. 2), suggesting that CRP on POD 3 could be useful to predict postoperative infective complications. Therefore, these results are consistent with those reported by Platt et al.

A limitation of this study is that retrospective data collection relied only on evaluation of clinical progress notes, laboratory test results, and other documentation. However, three-quarters of patients in this study were older than 65 years of age. Therefore, we believe our results apply to the elderly, in whom serum albumin is likely decreased. Prospective studies are needed to confirm whether our findings can be adapted to all colorectal surgery patients.

## Conclusions

The present study revealed that CRP has the greatest association with serum albumin in the preoperative period and on PODs 3 and 7 and that antecedent CRP was associated with future serum albumin. Additionally, CRP on POD 3 could be useful in predicting hypoalbuminemia on POD 7. This result suggests that CRP on POD 3 may be valuable as an indicator of early nutritional intervention.

## Abbreviations

AJCC: American Joint Committee on Cancer
ALP: alkaline phosphatase
ALT: alanine aminotransferase
AST: aspartate aminotransferase
AUC: the area under the receiver operating characteristic curve
BMI: body mass index
BUN: blood urea nitrogen
CRP: C-reactive protein
γ-GTP: gamma-glutamyl transpeptidase
Hb: hemoglobin
LDH: lactate dehydrogenase
POD: postoperative day
ROC: receiver operating characteristic
Scr: serum creatinine
TNM: tumor-node-metastasis
WBC: white blood cell

## References

[1] Alves A, Panis Y, Mathieu P (2005). Postoperative mortality and morbidity in french patients undergoing colorectal surgery: results of a prospective multicenter study. Arch Surg.

[2] Abraham N (2011). Enhanced recovery after surgery programs hasten recovery after colorectal resections. World J Gastrointest Surg.

[3] Ramírez JM, Blasco JA, Roig JV, Maeso-Martínez S, Casal JE, Esteban F, Lic DC (2011). Enhanced recovery in colorectal surgery: a multicentre study. BMC Surg.

[4] Platt JJ, Ramanathan ML, Crosbie RA, Anderson JH, McKee RF, Horgan PG, McMillan DC (2012). C-reactive protein as a predictor of postoperative infective complications after curative resection in patients with colorectal cancer. Ann Surg Oncol.

[5] Fujita S, Saito N, Yamada T (2007). Randomized, multicenter trial of antibiotic prophylaxis in elective colorectal surgery: single dose vs 3 doses of a second-generation cephalosporin without metronidazole and oral antibiotics. Arch Surg.

[6] Fleck A, Raines G, Hawker F, Trotter J, Wallace PI, Ledingham IM, Calman KC (1985). Increased vascular permeability: a major cause of hypoalbuminaemia in disease and injury. Lancet.

[7] Nicholson JP, Wolmarans MR, Park GR (2000). The role of albumin in critical illness. Br J Anaesth.

[8] Gibbs J, Cull W, Henderson W, Daley J, Hur K, Khuri SF (1999). Preoperative serum albumin level as a predictor of operative mortality and morbidity: results from the national va surgical risk study. Arch Surg.

[9] Franch-Arcas G (2001). The meaning of hypoalbuminaemia in clinical practice. Clin Nutr Edinb Scotl.

[10] Leite HP, Fisberg M, de Carvalho WB, de Camargo Carvalho AC (2005). Serum albumin and clinical outcome in pediatric cardiac surgery. Nutr Burbank Los Angel Cty Calif.

[11] Lohsiriwat V, Chinswangwatanakul V, Lohsiriwat S, Akaraviputh T, Boonnuch W, Methasade A, Lohsiriwat D (2007). Hypoalbuminemia is a predictor of delayed postoperative bowel function and poor surgical outcomes in right-sided colon cancer patients. Asia Pac J Clin Nutr.

[12] Lohsiriwat V (2008). Pre-operative hypoalbuminemia is a major risk factor for postoperative complications following rectal cancer surgery. World J Gastroenterol.

[13] Bhamidipati CM, LaPar DJ, Mehta GS, Kern JA, Upchurch GR, Kron IL, Ailawadi G (2011). Albumin is a better predictor of outcomes than body mass index following coronary artery bypass grafting. Surgery.

[14] Weimann A, Braga M, Harsanyi L, Laviano A, Ljungqvist O, Soeters P, DGEM (German Society for Nutritional Medicine), Jauch KW, Kemen M, Hiesmayr JM, Horbach T, Kuse ER, Vestweber KH, ESPEN (European Society for Parenteral and Enteral Nutrition). ESPEN Guidelines on Enteral Nutrition: Surgery including organ transplantation. Clin Nutr Edinb Scotl 2006; 25:224–44. 10.1016/j.clnu.2006.01.015 16698152

[15] Welsch T, Müller SA, Ulrich A, Kischlat A, Hinz U, Kienle P, Büchler MW, Schmidt J, Schmied BM (2007). C-reactive protein as early predictor for infectious postoperative complications in rectal surgery. Int J Colorectal Dis.

[16] Matthiessen P, Henriksson M, Hallböök O, Grunditz E, Norén B, Arbman G (2008). Increase of serum C-reactive protein is an early indicator of subsequent symptomatic anastomotic leakage after anterior resection. Colorectal Dis Off J Assoc Coloproctol G B Irel.

[17] Kørner H, Nielsen HJ, Søreide JA, Nedrebø BS, Søreide K, Knapp JC (2009). Diagnostic accuracy of C-reactive protein for intraabdominal infections after colorectal resections. J Gastrointest Surg Off J Soc Surg Aliment Tract.

[18] Woeste G, Müller C, Bechstein WO, Wullstein C (2010). Increased serum levels of C-reactive protein precede anastomotic leakage in colorectal surgery. World J Surg.

[19] Ortega-Deballon P, Radais F, Facy O, d’ Athis P, Masson D, Charles PE, Cheynel N, Favre JP, Rat P (2010). C-reactive protein is an early predictor of septic complications after elective colorectal surgery. World J Surg.

[20] MacKay GJ, Molloy RG, O’Dwyer PJ (2011). C-reactive protein as a predictor of postoperative infective complications following elective colorectal resection. Colorectal Dis Off J Assoc Coloproctol G B Irel.

[21] Warschkow R, Tarantino I, Torzewski M, Näf F, Lange J, Steffen T (2011). Diagnostic accuracy of C-reactive protein and white blood cell counts in the early detection of inflammatory complications after open resection of colorectal cancer: a retrospective study of 1,187 patients. Int J Colorectal Dis.

[22] Al-Shaiba R, McMillan DC, Angerson WJ, Leen E, McArdle CS, Horgan P (2004). The relationship between hypoalbuminaemia, tumour volume and the systemic inflammatory response in patients with colorectal liver metastases. Br J Cancer.

[23] Feng J-F, Zhao Q, Chen Q-X (2014). Prognostic significance of Glasgow prognostic score in patients undergoing esophagectomy for esophageal squamous cell carcinoma. Saudi J Gastroenterol Off J Saudi Gastroenterol Assoc.

[24] Hashiguchi Y, Hase K, Kotake K, Ueno H, Shinto E, Mochizuki H, Yamamoto J, Sugihara K (2012). Evaluation of the seventh edition of the tumour, node, metastasis (TNM) classification for colon cancer in two nationwide registries of the United States and Japan. Colorectal Dis Off J Assoc Coloproctol G B Irel.

[25] Moyes LH, Leitch EF, McKee RF, Anderson JH, Horgan PG, McMillan DC (2009). Preoperative systemic inflammation predicts postoperative infectious complications in patients undergoing curative resection for colorectal cancer. Br J Cancer.

[26] Vincent J-L, Russell JA, Jacob M, Martin G, Guidet B, Wernerman J, Roca RF, McCluskey SA, Gattinoni L (2014). Albumin administration in the acutely ill: what is new and where next?. Crit Care.

[27] Dong R, Guo Z-Y (2010). Gastrointestinal symptoms in patients undergoing peritoneal dialysis: multivariate analysis of correlated factors. World J Gastroenterol WJG.

[28] El Khouli RH, Macura KJ, Barker PB, Habba MR, Jacobs MA, Bluemke DA (2009). Relationship of temporal resolution to diagnostic performance for dynamic contrast enhanced MRI of the breast. J Magn Reson Imaging JMRI.

[29] Kanda Y (2013). Investigation of the freely available easy-to-use software “EZR” for medical statistics. Bone Marrow Transplant.

[30] Oberhofer D, Juras J, Pavicić AM, Rancić Zurić I, Rumenjak V (2012). Comparison of C-reactive protein and procalcitonin as predictors of postoperative infectious complications after elective colorectal surgery. Croat Med J.

[31] Silvestre J, Rebanda J, Lourenço C, Póvoa P (2014). Diagnostic accuracy of C-reactive protein and procalcitonin in the early detection of infection after elective colorectal surgery—a pilot study. BMC Infect Dis.

[32] Gabay C, Kushner I (1999). Acute-phase proteins and other systemic responses to inflammation. N Engl J Med.

[33] Singh PP, Zeng ISL, Srinivasa S, Lemanu DP, Connolly AB, Hill AG (2014). Systematic review and meta-analysis of use of serum C-reactive protein levels to predict anastomotic leak after colorectal surgery. Br J Surg.

